# Hydatid pulmonary arterial embolization from an intracardiac cyst mimicking pulmonary thromboembolism: A case report

**DOI:** 10.1016/j.rmcr.2026.102510

**Published:** 2026-09-28

**Authors:** Abdulhay Tannari, Mohamad Alrajab, Wael Mahmood, Ahmad Sheikh Mohammad, Noura Abdulkader, Abdullah Al Yahya, Abdulraouf Haj Yousef, Mustafa Alyusuf, Ahmad Alhasan

**Affiliations:** a Department of Internal Medicine, Pulmonology Division, Idlib University Hospital, Idlib, Syria; b Department of Internal Medicine, Idlib University Hospital, Idlib, Syria; c Department of Radiology, Idlib University Hospital, Idlib, Syria; d Department of Cardiology, Idlib University Hospital, Idlib, Syria

**Keywords:** Hydatid disease, Pulmonary artery, Pulmonary embolism mimic, Cardiac hydatid cyst, *Echinococcus granulosus*

## Abstract

**Background:**

Cystic echinococcosis most commonly affects the liver and lungs. Cardiac involvement is uncommon, and embolization to the pulmonary arteries is exceptionally rare. Such presentations may closely mimic pulmonary embolism, leading to diagnostic delay.

**Case presentation:**

A 14-year-old boy from an endemic rural region presented with progressive dyspnea, intermittent chest pain, recurrent hemoptysis, and severe resting hypoxemia, with an oxygen saturation of 85% on room air. CT pulmonary angiography demonstrated extensive bilateral pulmonary arterial filling defects with cystic morphology, associated arterial dilatation, and representative attenuation values of approximately −5 HU. Despite the extensive vascular obstruction, D-dimer was only mildly elevated at 0.83 mg/L FEU (reference range, 0–0.5 mg/L FEU), compared with 0.41 mg/L FEU during a clinically stable period. Transthoracic echocardiography identified an approximately 22 × 22 mm right-sided intracardiac cyst adjacent to the tricuspid valve, and *Echinococcus* IgG ELISA was positive at 32 U/L (positive threshold, >11 U/L). The patient underwent median sternotomy with cardiopulmonary bypass, intracardiac cyst excision, and bilateral pulmonary arteriotomy, with removal of 18 large pulmonary arterial cystic structures. Follow-up CT pulmonary angiography at 6 months demonstrated marked regression of the intravascular disease burden, with small residual distal lesions. However, repeat CT approximately 12 months after surgery demonstrated an approximately 15–20% visually estimated increase in bilateral pulmonary arterial cystic burden, with new proximal pulmonary arterial cystic lesions, consistent with interval radiological progression. This progression was documented after discontinuation of albendazole, although a causal relationship could not be established.

**Conclusion:**

Pulmonary arterial hydatid embolization should be considered when presumed pulmonary thromboembolism is accompanied by discordant laboratory and imaging findings, particularly in endemic regions. In this patient, extensive bilateral fluid-attenuation intravascular lesions, only mildly elevated D-dimer despite severe obstruction, and a concomitant intracardiac cyst supported a non-thrombotic embolic process. Combined surgical and antiparasitic treatment resulted in marked initial radiological improvement; however, subsequent progression of residual pulmonary arterial disease highlights the need for prolonged radiological surveillance and careful reassessment of the duration of antiparasitic therapy.

## LEARNING POINTS

• Pulmonary arterial hydatid disease is an important mimic of pulmonary embolism in endemic regions.
• Low-attenuation cystic filling defects with only mildl D-dimer elevation should prompt reconsideration of thromboembolism.
• A concomitant intracardiac cyst may identify the embolic source and guide multidisciplinary management.

## 1 Introduction

Cystic echinococcosis, caused by *Echinococcus granulosus*, is a parasitic zoonosis in which humans act as accidental intermediate hosts, most commonly affecting the liver and lungs [1,2].

Involvement of the heart and vessels is infrequent but significant due to the potential for embolization and serious complications [1,3]. Pulmonary artery involvement is particularly rare and may occur secondary to embolization from a cardiac hydatid cyst or systemic dissemination [4].

Intravascular hydatid cysts can produce clinical and radiological findings that resemble pulmonary embolism and contribute to diagnostic delay [5]. In endemic regions, where transmission between dogs, livestock, and humans persists, this overlap may further increase the risk of misdiagnosis [2,6].

## 2 Case description

A 14-year-old boy from a rural area of northwestern Syria presented with progressive dyspnea, intermittent chest pain, and recurrent hemoptysis that began in mid-February 2025. Because of displacement during the conflict, his movements were limited to rural areas within northern Syria, with no history of international travel. He had prolonged household exposure to dogs in a rural livestock-raising environment. He had no known chronic medical conditions, previous surgery, or drug or food allergies.

According to the available outside-facility records, pulmonary thromboembolism was initially suspected, and oral rivaroxaban was initiated on 13 April 2025 and discontinued on 17 May 2025.

However, his symptoms progressively worsened despite anticoagulant therapy, prompting admission to Idlib University Hospital in Syria on 13 May 2025 with worsening dyspnea, recurrent hemoptysis, and severe hypoxemia (oxygen saturation 85% on room air).

Chest radiography demonstrated bilateral perihilar and basal opacities (Fig. 1).

**Fig. 1:**
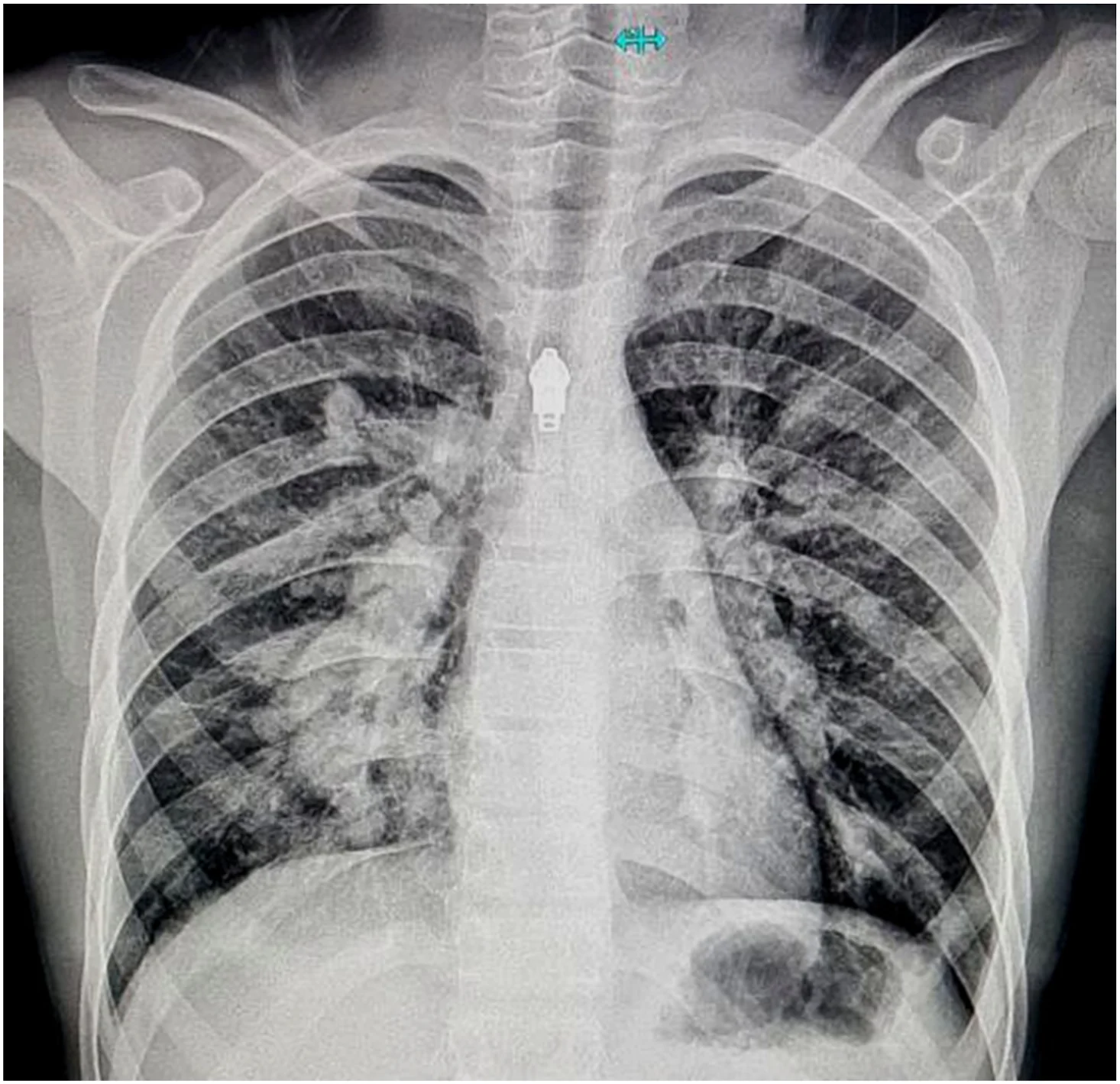
Chest radiograph showing bilateral perihilar opacities.

Laboratory testing showed a WBC count of 8.7 × 10^3^/μL, hemoglobin of 12.9 g/dL, platelet count of 423 × 10^3^/μL, C-reactive protein of 64 mg/L, erythrocyte sedimentation rate of 22 mm/h, and mildly elevated D-dimer of 0.83 mg/L FEU during the acute symptomatic episode (reference range, 0–0.5 mg/L FEU). D-dimer subsequently decreased to 0.41 mg/L FEU during a clinically stable period. Autoimmune testing was non-diagnostic: ANA, anti-GBM antibodies, and PR3-ANCA were negative, while MPO-ANCA was only marginally above the laboratory cutoff, without clinical evidence of systemic vasculitis. A multiplex thrombophilia PCR panel was negative for factor V Leiden and prothrombin G20210A mutations. Heterozygous MTHFR C677T and PAI-1 4G variants were detected but were not considered clinically explanatory for the extensive pulmonary arterial abnormalities.

Axial CT imaging revealed multiple intraluminal filling defects within the pulmonary arteries and a right-sided intracardiac cystic lesion (Fig. 2). Transthoracic echocardiography demonstrated an approximately 22 × 22 mm right-sided cystic lesion adjacent to the tricuspid valve, with a left ventricular ejection fraction of approximately 55% and no pericardial effusion. The available echocardiography report did not document lesion mobility, a precise attachment site, quantitative right ventricular function, or estimated pulmonary artery pressure.

**Fig. 2:**
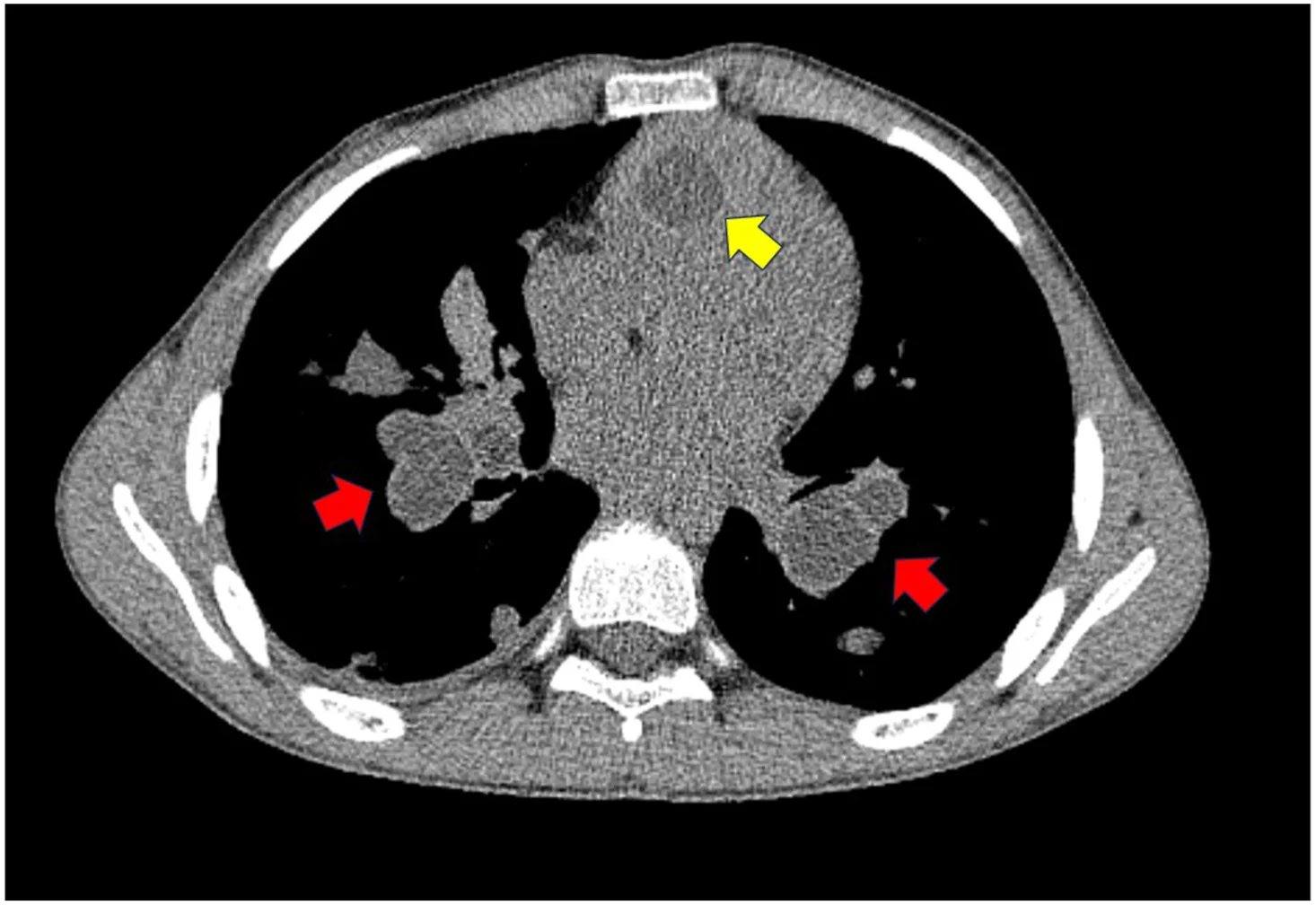
Axial CTPA demonstrating multiple intraluminal low-attenuation filling defects within the pulmonary arterial branches (red arrows), with an associated right-sided intracardiac cystic lesion (yellow arrow).

CT pulmonary angiography demonstrated marked dilatation of the pulmonary arteries with multiple cystic intraluminal filling defects (Fig. 3A–C). Attenuation measurements obtained from representative intravascular lesions were approximately −5 HU, supporting fluid-containing cystic material rather than conventional thromboembolic material (Fig. 4).

**Fig. 3:**
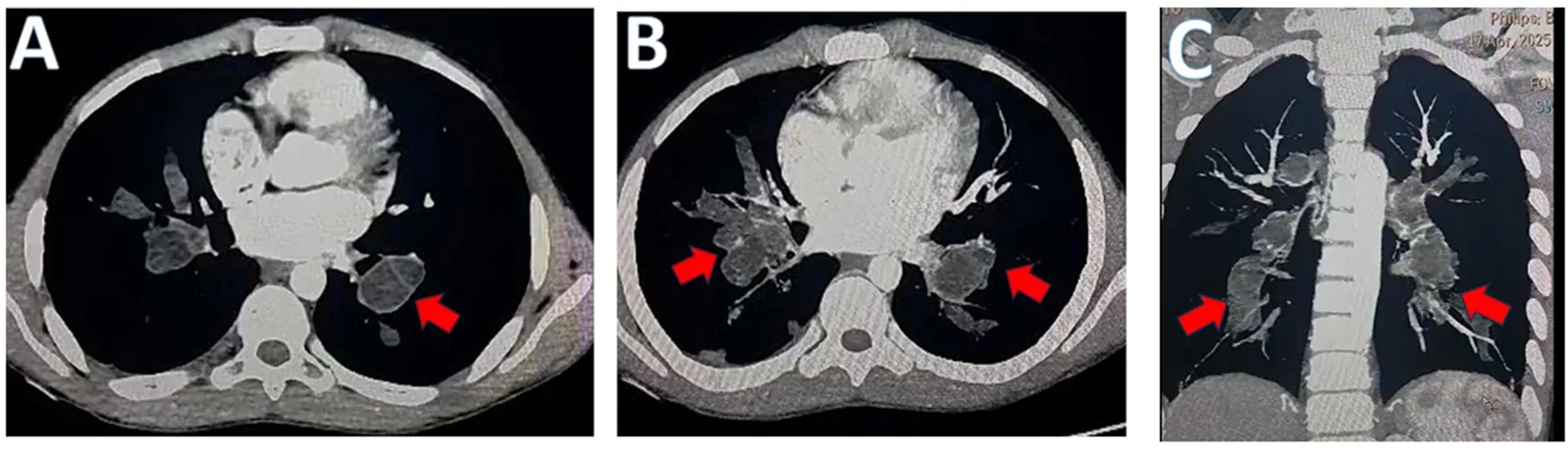
A–C. CT pulmonary angiography. (A, B) Axial images showing multiple intraluminal low-attenuation filling defects (arrows) within the right and left pulmonary arterial branches, associated with arterial dilatation. (C) Coronal MPR image demonstrating the extent of intraluminal involvement (arrows).

**Fig. 4:**
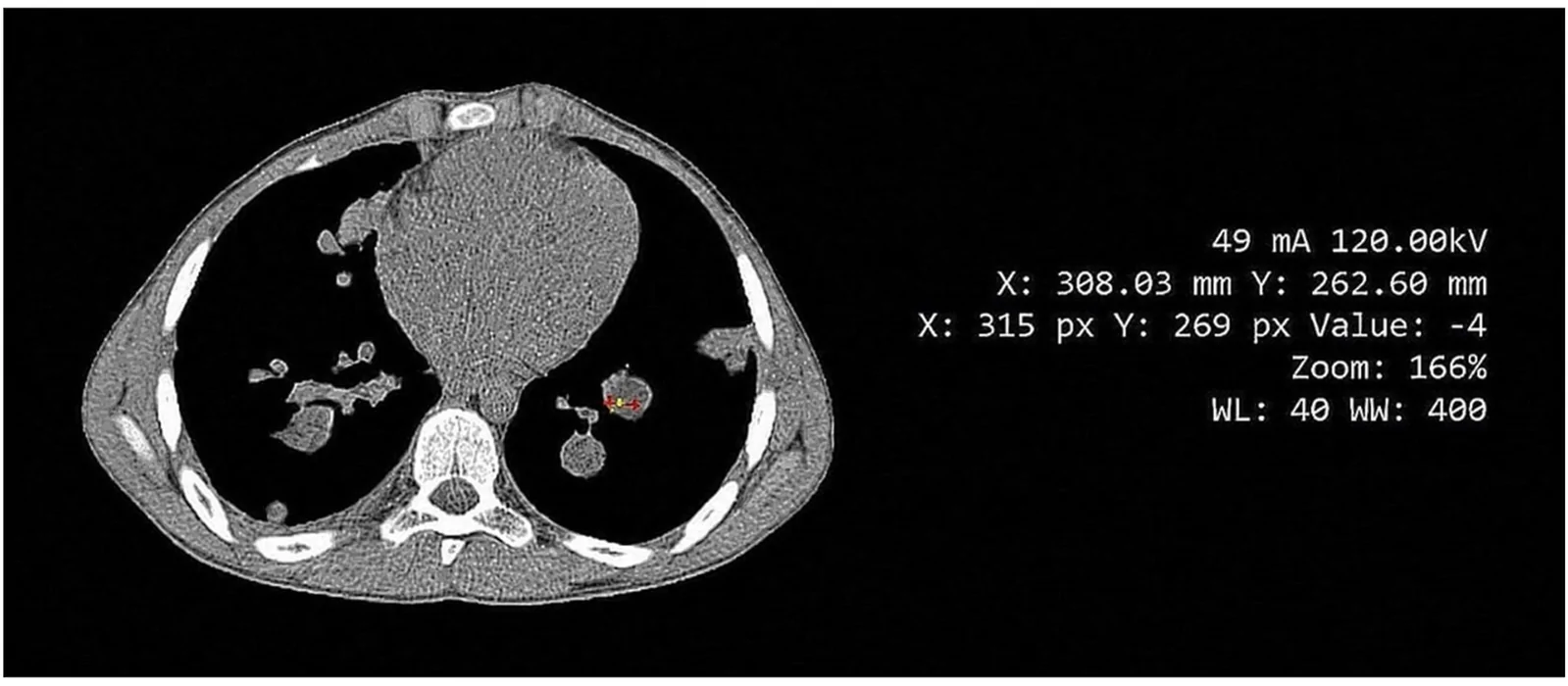
Non-contrast axial chest CT demonstrating a representative low-attenuation intraluminal pulmonary arterial lesion, with an attenuation measurement of −4 HU, supporting its fluid-containing cystic nature.

On 17 May 2025, the combination of a mildly elevated D-dimer level, disproportionate to the extensive bilateral pulmonary arterial obstruction, and the imaging finding of an intracardiac cyst raised suspicion for pulmonary hydatid embolization. Consequently, the initial diagnosis of pulmonary thromboembolism was reconsidered, and anticoagulation was discontinued. The diagnosis was further supported by a positive *Echinococcus* IgG ELISA result (32 U/L; positive threshold >11 U/L) and a striking family history: the patient's mother, sister, and brother had hydatid disease, and several other affected relatives had previously undergone hepatic hydatid cyst surgery. The family also reported prolonged exposure to dogs, sheep, and other livestock while living in rural northern Syria.

Following diagnostic confirmation in mid-May 2025, albendazole 400 mg twice daily was initiated to reduce cyst viability and the risk of further dissemination [7,8].

Given the patient's progressive dyspnea, recurrent episodes of hemoptysis, hypoxemia, extensive bilateral pulmonary arterial involvement on CT pulmonary angiography, and the presence of an intracardiac hydatid cyst on echocardiography, the patient was referred to a tertiary care center abroad for surgical management due to limited local resources.

On 4 July 2025, the patient underwent median sternotomy under general anesthesia. Cardiopulmonary bypass was established using aortic and bicaval cannulation. A right atriotomy revealed an intracardiac cyst in close relation to the tricuspid valve apparatus. The cyst was carefully dissected and excised while preserving the valve. Pulmonary arteriotomy was then extended into the right and left pulmonary arteries, and 18 large cystic structures were removed bilaterally (Fig. 5). The pulmonary arteriotomy and right atriotomy were closed, the heart was carefully de-aired, and the patient was successfully weaned from cardiopulmonary bypass in sinus rhythm with good cardiac output. The operative report did not document intraoperative cyst rupture, use of a scolicidal solution, or a specific rupture-management protocol. Although histopathological confirmation was unavailable, the diagnosis was strongly supported by convergent imaging, serological, epidemiological, and operative findings, including an intracardiac source and retrieval of gross cystic material.

**Fig. 5:**
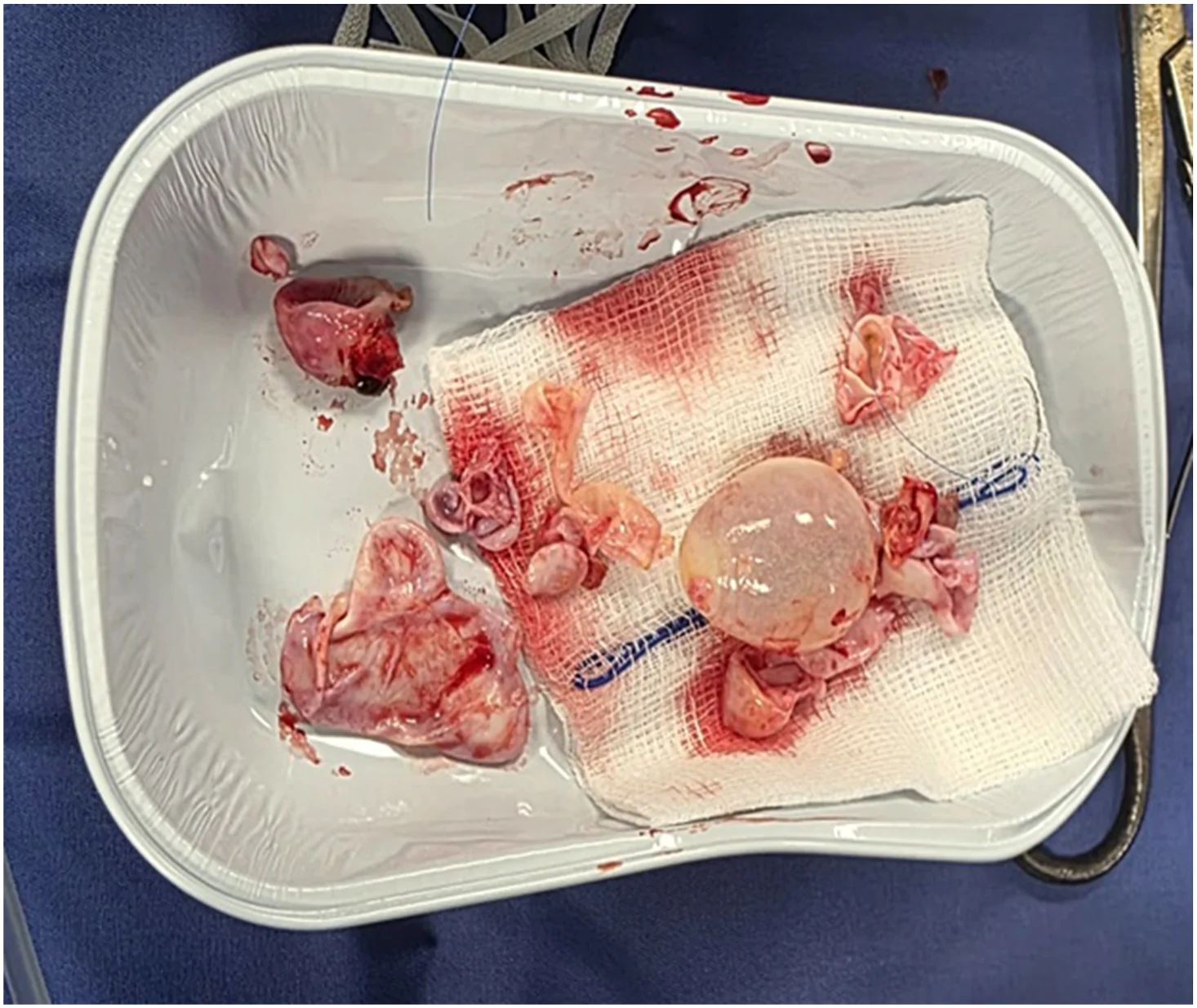
Gross surgical specimen demonstrating excised hydatid cyst material removed during surgery.

The postoperative course was generally uneventful. Mild intermittent hemoptysis persisted during the early postoperative period but resolved completely within a few weeks. Albendazole was administered postoperatively. Although prolonged therapy had been recommended by our team, the patient discontinued albendazole after 3 months following advice from the treating surgeon. Postoperative anticoagulation was documented, but the agent and duration were unavailable in the records reviewed. Follow-up CTPA at 6 months demonstrated an approximately 70% visually estimated reduction in intraluminal cystic burden, with persistence of smaller distal cystic lesions (Fig. 6A and B).

**Fig. 6:**
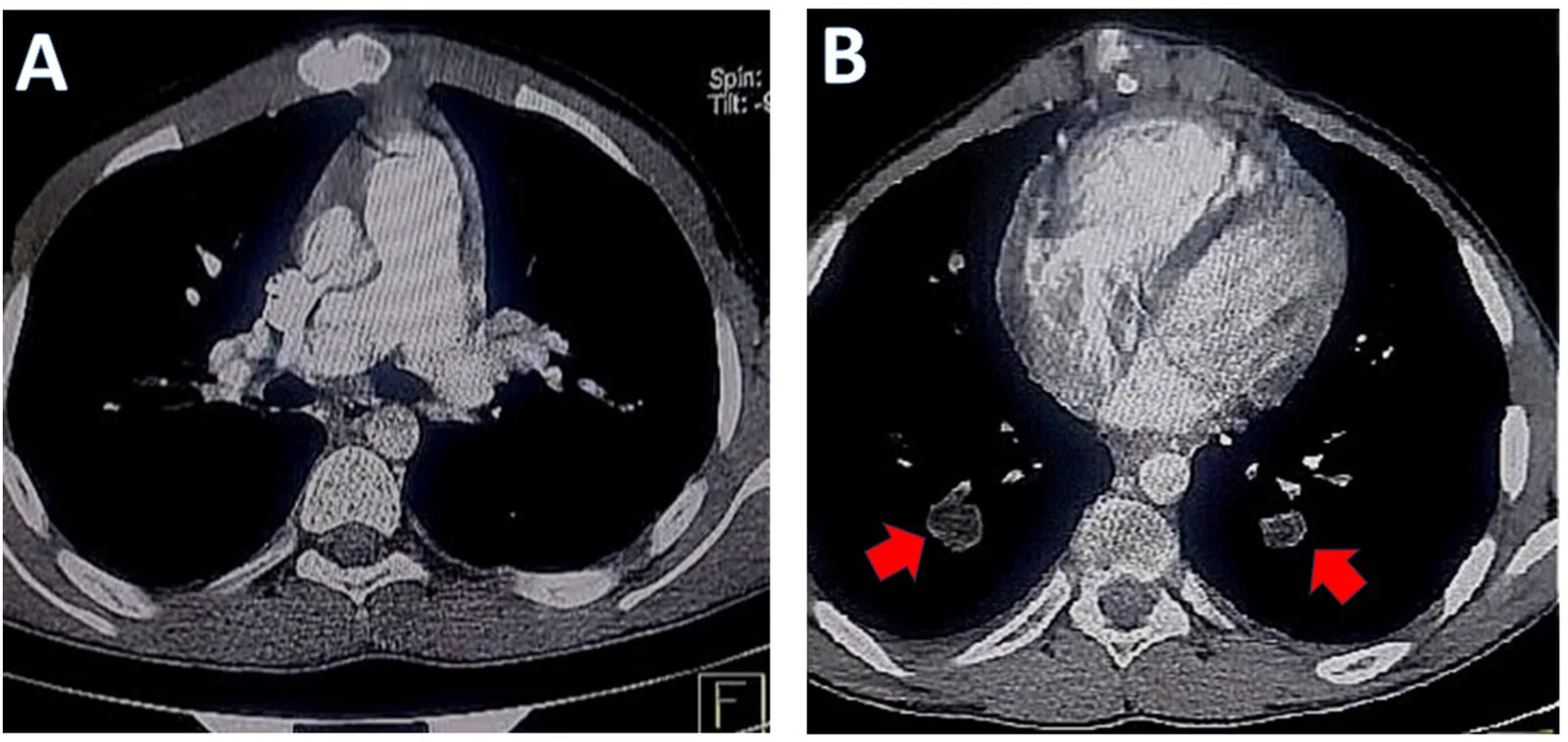
A–B. Follow-up CTPA images 6 months after surgical removal of the intracardiac and pulmonary arterial hydatid cysts. (A) Axial image demonstrating marked reduction of intraluminal cystic lesions. (B) Axial image demonstrating persistence of smaller cystic lesions in distal pulmonary arteries (arrow).

At the most recent follow-up, approximately 12 months after surgery, the patient was asymptomatic and had resumed normal school attendance and daily activities. Transthoracic echocardiography at that time demonstrated moderate right-sided chamber dilatation, mild tricuspid regurgitation, reduced tricuspid annular plane systolic excursion (TAPSE, approximately 10 mm), preserved left ventricular systolic function (LVEF approximately 60%), an estimated pulmonary artery systolic pressure of 27 mmHg, and no pericardial effusion.

Repeat CT performed approximately 12 months after surgery (Fig. 7A and B) demonstrated an approximately 15–20% visually estimated interval increase in bilateral pulmonary arterial cystic burden compared with the 6-month follow-up examination. Whereas the earlier study showed marked regression with residual lesions predominantly confined to the basal pulmonary arterial branches, the later examination revealed two new proximal cystic lesions near the origin of the pulmonary artery, measuring approximately 20 mm, together with multiple additional bilateral lesions measuring up to 25 mm on the right and 23 mm on the left, with extension into distal pulmonary arterial branches. These findings were consistent with interval radiological progression.

**Fig. 7:**
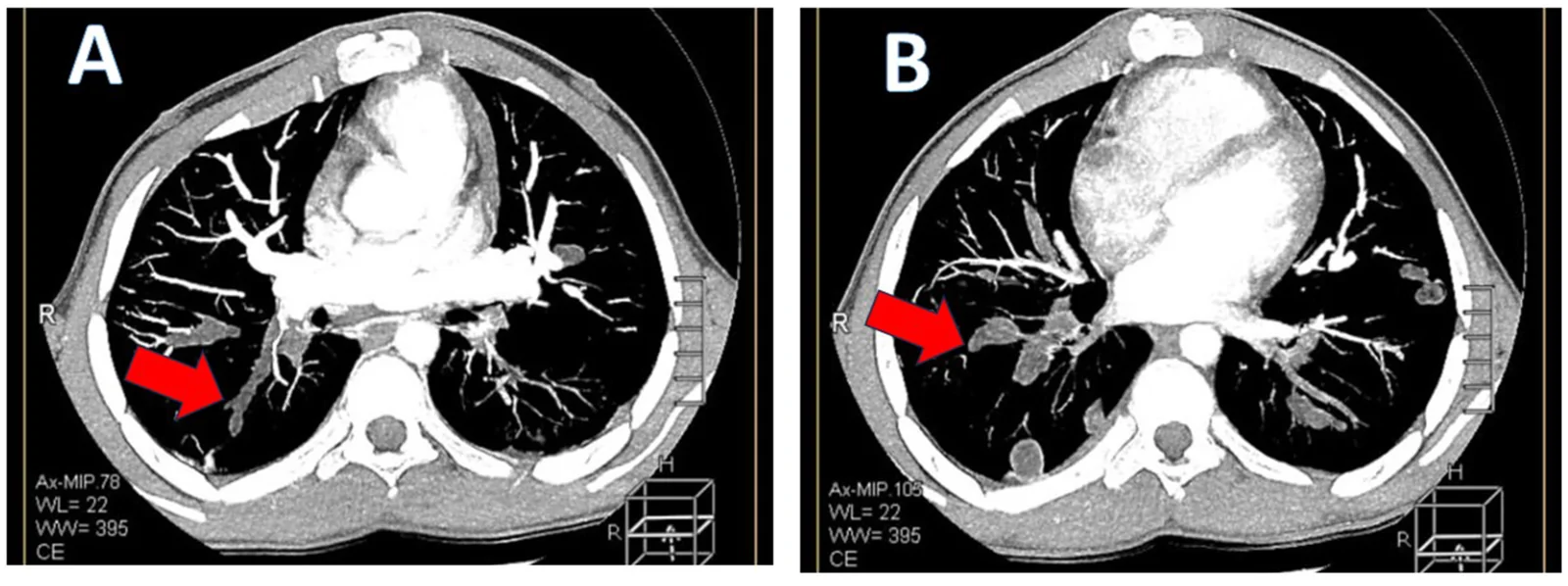
A–B. Follow-up CT approximately 12 months after surgery showing bilateral pulmonary arterial cystic lesions; comparison with the 6-month examination demonstrated an interval increase in cystic burden. (A) Axial maximum-intensity-projection image showing persistent cystic filling defects within the right lower-lobe pulmonary arterial branches (red arrow). (B) A more caudal axial image demonstrating multiple bilateral pulmonary arterial cystic lesions, more extensive on the right, with persistent distal involvement (red arrow).

A graphical timeline summarizing the patient's clinical course, diagnostic evaluation, treatment, and follow-up is presented in Fig. 8.

**Fig. 8:**
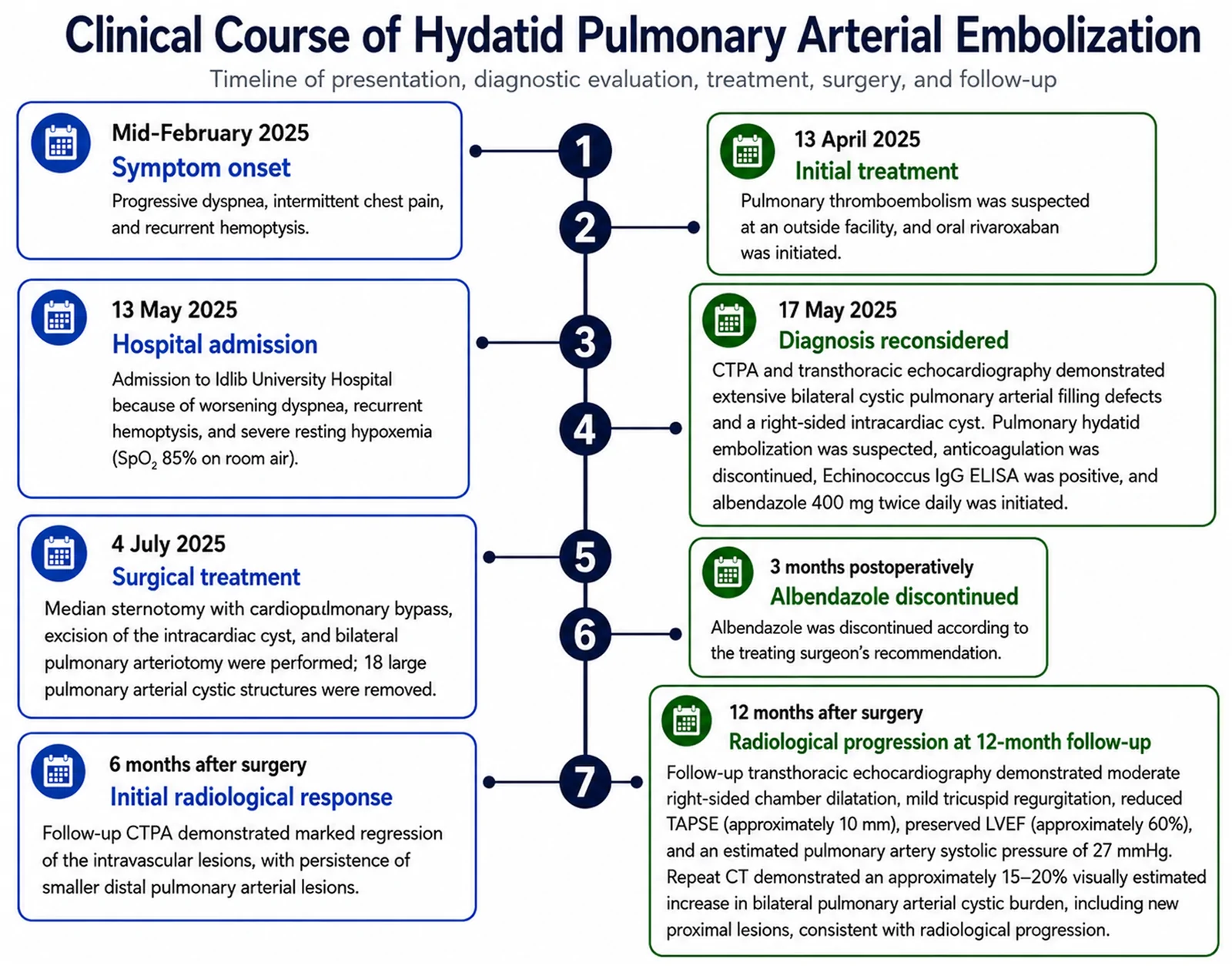
Graphical timeline of the patient's clinical course, including presentation, diagnostic reassessment, treatment, surgery, and follow-up at 6 and 12 months.

## 3 Discussion

Hydatid disease is a zoonotic infection that may remain asymptomatic for years but can lead to severe complications when cardiac or vascular structures are involved [1,3]. Cardiovascular involvement is uncommon, and pulmonary artery involvement represents a rare manifestation that may occur secondary to embolization or extension of intracardiac cysts [1,4].

Pulmonary artery hydatid disease poses a significant diagnostic challenge because it can closely mimic acute pulmonary thromboembolism [5,8]. In our patient, the key discordant findings were fluid-attenuation intravascular lesions, with representative attenuation measurements of approximately −5 HU, only mildly elevated D-dimer despite extensive bilateral obstruction, and a concomitant right-sided intracardiac cyst. The limited fluctuation in D-dimer—from 0.41 mg/L FEU during a clinically stable period to 0.83 mg/L FEU during the acute symptomatic episode—remained disproportionate to the extensive bilateral pulmonary arterial obstruction, supporting a non-thrombotic embolic process.

Comparison with previously reported cases further clarifies the contribution of the present report. Odev et al. described pulmonary embolization secondary to cardiac hydatid disease and highlighted the imaging features of this unusual complication [4]. More recently, Bouhamdi et al. reported hydatid pulmonary embolism arising from a right ventricular hydatid cyst in a 23-year-old patient for whom surgery was considered infeasible and medical therapy was administered [8]. In contrast, our patient was 14 years old and underwent combined surgical excision of the intracardiac cyst and bilateral removal of pulmonary arterial cystic lesions. Although marked radiological regression was observed at 6 months, interval progression of residual pulmonary arterial disease was documented at approximately 12 months.

The present case is distinguished by several clinically relevant features: pediatric presentation; extensive bilateral pulmonary arterial involvement; quantitative measurement of intravascular lesion attenuation at approximately −5 HU; only mildly elevated D-dimer despite severe vascular obstruction; operative removal of 18 large bilateral pulmonary arterial cystic structures under cardiopulmonary bypass; and longitudinal imaging demonstrating marked initial regression followed by interval progression of residual disease. Thus, the novelty of this report lies not in being the first description of cardiac-origin hydatid embolization, but in the integrated documentation of its pediatric presentation, quantitative imaging features, operative findings, epidemiological context, and longitudinal outcome.

The epidemiological history suggested local acquisition in rural northwestern Syria, an endemic livestock-raising region where the patient and multiple affected family members had prolonged exposure to dogs, sheep, and other livestock. The clustering of hydatid disease within the household, including hepatic hydatid disease requiring surgery in several relatives, suggests a shared environmental source of exposure—possibly through contaminated food or water or contact with infected dogs—rather than person-to-person transmission. Humans acquire infection by ingesting *Echinococcus* eggs shed in canine feces, either directly or through contaminated food, water, soil, or hands [1,2].

After intestinal penetration, oncospheres generally reach the liver and lungs; cardiac involvement may occur through coronary circulation, pulmonary veins, lymphatic pathways, or systemic dissemination. In this patient, growth of a right-sided intracardiac cyst adjacent to the tricuspid valve may have resulted in repeated embolization of daughter cysts or hydatid material into the pulmonary arterial circulation. The residual distal lesions observed on postoperative follow-up imaging may represent pre-existing cystic emboli in peripheral pulmonary arterial branches that were inaccessible to surgical removal.

Imaging was crucial for characterizing the lesions. On CT, the intraluminal filling defects showed low attenuation (approximately −5 HU), favoring cystic content over conventional thromboembolic material [5]. The concomitant intracardiac cyst further supported a parasitic origin and suggested a cardiac source of pulmonary arterial embolization. Accordingly, the complementary use of CT pulmonary angiography and transthoracic echocardiography enabled identification of both the pulmonary arterial lesions and their probable intracardiac source [5,7].

Surgical removal remains the cornerstone of treatment, particularly in cases with significant vascular obstruction [3]. However, complete clearance may be limited by distal or inaccessible lesions, as observed in our patient, in whom residual distal disease persisted postoperatively. Although cyst rupture was not documented in the available operative report, it cannot be excluded and may have contributed to distal dissemination and persistence of peripheral pulmonary arterial lesions. In this context, adjunctive albendazole therapy plays an important role in reducing cyst viability and minimizing the risk of recurrence [7].

Despite clinical recovery and a normal estimated pulmonary artery systolic pressure at follow-up, moderate right-sided chamber dilatation and reduced TAPSE suggested persistent right ventricular remodeling or dysfunction, warranting continued cardiology surveillance. Follow-up CTPA at 6 months demonstrated marked regression of the pulmonary arterial cystic burden, with residual lesions predominantly confined to the basal pulmonary arterial branches (Fig. 6A and B). Repeat CT approximately 12 months after surgery demonstrated an approximately 15–20% visually estimated increase in bilateral pulmonary arterial cystic burden compared with the 6-month examination. Two new proximal cystic lesions measuring approximately 20 mm were identified, together with multiple bilateral lesions measuring up to 25 mm on the right and 23 mm on the left, with extension into distal pulmonary arterial branches (Fig. 7A and B). These findings were consistent with interval radiological progression. Progression was documented after albendazole had been discontinued at 3 months postoperatively. Although this temporal association does not establish causality, it raises the possibility that a 3-month postoperative course may have been insufficient in the setting of residual or surgically inaccessible disease. The optimal duration of antiparasitic therapy in this setting remains uncertain.

This case also highlights the One Health implications of cystic echinococcosis in rural livestock-raising communities. Continued transmission may be facilitated by close dog–livestock contact, unsafe disposal of infected offal, and inadequate canine deworming. Control therefore requires coordinated veterinary and public-health measures, including regular praziquantel treatment of dogs, safe disposal of infected viscera, improved slaughter hygiene, and community education [2,9]. The strong household clustering and prolonged livestock exposure in our patient further emphasize the importance of maintaining clinical suspicion in endemic settings.

## CRediT authorship contribution statement

**Abdulhay Tannari:** Writing – review & editing, Writing – original draft, Supervision, Project administration, Methodology, Investigation, Conceptualization. **Mohamad Alrajab:** Writing – review & editing, Validation, Investigation, Data curation. **Wael Mahmood:** Writing – review & editing, Visualization, Validation, Investigation. **Ahmad Sheikh Mohammad:** Writing – review & editing, Validation, Investigation. **Noura Abdulkader:** Writing – review & editing, Validation, Investigation, Data curation. **Abdullah Al Yahya:** Writing – review & editing, Investigation, Data curation. **Abdulraouf Haj Yousef:** Writing – review & editing, Validation, Investigation. **Mustafa Alyusuf:** Data curation, Investigation, Writing – review & editing. **Ahmad Alhasan:** Writing – review & editing, Validation, Supervision, Investigation, Data curation.

## Declaration of generative AI and AI-assisted technologies in the manuscript preparation process

During the preparation of this work, the authors used ChatGPT (OpenAI) to assist with language editing and improving the clarity of the manuscript. The authors reviewed and edited the output as needed and take full responsibility for the content of the article.

## Declaration of competing interest

The authors declare that they have no known competing financial interests or personal relationships that could have appeared to influence the work reported in this paper.
